# Family outcomes of a parent-implemented early intervention for neurodevelopmental disorders: an exploratory study of family dynamics

**DOI:** 10.3389/fpubh.2025.1614089

**Published:** 2025-07-08

**Authors:** Cecilia H. M. Wong, Camille K. Y. Chan, Paul W. C. Wong

**Affiliations:** ^1^Faculty of Social Sciences, University of Hong Kong, Pokfulam, Hong Kong SAR, China; ^2^Department of Social Work and Social Administration, Faculty of Social Sciences, University of Hong Kong, Pokfulam, Hong Kong SAR, China

**Keywords:** neurodiversity, neurodevelopemntal disorders, parent-implemented early intervention program, autism spectral disorder (ASD), caregiver training, family dynamics, program evaluation, impact assessment (IA)

## Abstract

**Background:**

Neurodevelopmental disorders have become global public health challenges, and early interventions have been proved to be effective in mitigating the problems and promoting the long-term functioning of people facing such challenges. These interventions had long been provided only by health professionals, but parents are now recognized for their capabilities. Parent-implemented early interventions are devised to equip parents with specialized knowledge and skills so that they can offer tailored training for their children. As these community-based interventions are designed to be implemented at home, they inevitably influence and are influenced by the family systems. While the family dynamics play a key role in determining the efficacy of the intervention, still little is known about the familial factors, as the focus on the existing literature is on the changes of the parent–child dyads. This research bridged this knowledge gap in implementation sciences by investigating how the family members who did not partake in the training reacted to the parent-implemented interventions (PIIs) at home.

**Methods:**

A qualitative study was conducted between August 2023 and July 2024 in Hong Kong, examining the impacts of a localized version of the World Health Organization Caregiver Skills Training. In total, 22 respondents participated in five focus group discussions. Inductive reflexive thematic analysis was applied to construct codes, themes, and frameworks.

**Results:**

Four levels of responses—Level 0: Reject, Level 1: Support, Level 2: Attempt, and Level 3: Embrace—were identified, and these themes were on a continuum of families’ involvement in the practice of PII. Factors that promoted or discouraged their involvement were discussed. Evidence has confirmed that PII had indirect impacts on family dynamics, and the responses of family members affected the intervention effectiveness and the mental health of the caregiver-participants.

**Conclusion:**

This research responded to the call to improve public health evaluations. It moved beyond the linear changing processes in the intervention design, addressed the complexity of the social systems, and explicated the multidimensional changing processes in a family—the immediate context where parent-implemented early intervention was implemented. It contributed to build initial frameworks on the familial influences and support the future development of the intervention and research designs.

## 1 Background

Neurodevelopmental disorders have become public health problems that are of grave concern. The prevalence of developmental delay in low- and middle-income countries reached 18.8% as reported in a systematic review and meta-analysis published in 2024 (1), and the global prevalence of developmental disabilities among young children and adolescents stood at 7.2% as reported by the World Health Organization and the United Nations Children’s Fund (UNICEF) in 2023 (2). It is estimated that around the world, there are over 200 million children facing the challenges of developmental disabilities (3, 4).

For children with neurodevelopmental disorders, early interventions play a crucial role to set them on a better developmental trajectory and promote their long-term functioning (5, 6). While these interventions have long been delivered by health professionals, parents are now recognized for their potentials to partake in this service as they have more engagement time with their children (7, 8). Parent-implemented intervention (PII) is therefore devised to equip parents with specialized knowledge and skills so that they can provide training for their children at home. PII is not designed to replace the clinician-implemented interventions; it serves as a valuable resource to complement the professional services and support the families who are waiting for public services (9).

PII as a public health intervention is strategic. It recognizes family as a natural nurturing ground for children to learn and grow, and makes use of it to provide tailor-made learning opportunities (10, 11). More training opportunities yield more sustainable outcomes (12). Moreover, it positions parents as trainers and equips them with the capabilities to respond to their children’s needs and support them to reach their developmental milestones. This empowerment bolsters parents’ competencies, promotes their confidence and mental health, and equips them with skills to strengthen parent–child joint engagement (13–16).

In research that evaluated the effectiveness of PIIs, parents were found to be able to apply the learnt techniques, and they were confident to implement the interventions in the long run (9). Children with language and speech delay and children on the autism spectrum showed improvements in social communication skills and exhibited fewer challenging behaviors (12, 13, 17). Studies that compared PIIs with the clinician-implemented interventions reported that the results of PIIs were comparable to the professional services, and they even achieved better outcomes in some peripheral areas (7, 18). As an evidence-based intervention, PII is on the rise that draws attention from practitioners, researchers, and users (19).

Whereas studies have demonstrated the effectiveness of PIIs, the scope of the existing research was rather narrow that concentrated almost entirely on the changes of the parent-participants and their children. This focus is reasonable because PIIs aim at engendering positive changes in the parent–child dyads; through tracking their development, researchers can assess if the interventions have attained their goals. While we acknowledge the importance of this type of evaluation, we argue that widening the scope to examine the family dynamics that affect the efficacy of the interventions would unveil the protective and risk factors in families that are still missing in our implementation sciences. To date, there have been studies that touched on the impacts of PIIs on families; however, they have not yet offered an in-depth exploration of the changing mechanism and the positive and negative outcomes (20).

The exploration of the changes in the family system is indeed essential. Unlike the clinician-implemented interventions, which are usually conducted in a controlled environment such as a therapist’s room, PIIs are implemented in the family’s natural setting; thus they are inevitably influencing and influenced by the family dynamics. Only when we take the family context into consideration we can then construct a comprehensive understanding of the changing mechanisms and impacts of PIIs.

This stretching of scope to analyze the indirect impacts on other family members is also our response to the call for advancing the public health evaluations. The current evaluations largely focused on the linear changing processes of the direct participants, and there was an urgent need to expand our repertoire to capture the multidimensional changes in the families (21). Moreover, when complexity was addressed, the attention was usually on the complexity of the public health interventions; still, little is known about the complexity of the social systems (22). This study contributed to bridge this gap by examining the intricacies in the family social systems.

In this study, we investigated how family members who did not directly participate in the PII training responded to the implementation of the intervention at home. We adopted a qualitative research approach as this allowed us to delve into our respondents’ stories without being restricted by the predefined outcome measures that were commonly used in quantitative evaluation research. Meanwhile, as we aimed to fill the research gap of lacking insights into the changing processes in families, the qualitative method supported us to explore and collect data to develop preliminary frameworks that will contribute to both the professional practices and academic development.

## 2 Methods

### 2.1 The intervention

The PII studied in this research was the localized version of the World Health Organization Caregiver Skills Training (WHO-CST) (23, 24). WHO-CST was introduced to Hong Kong in 2018 as one of the services offered by the Jockey Club Autism Support Network program (JC A-Connect) (9). In 2021, the master trainers of WHO-CST in five local NGOs, namely Hip Hong Society, Hong Kong Young Women’s Christian Association, SAHK, The Salvation Army, and Tung Wah Group of Hospitals, worked together to further localize it to tailor to the professionals and caregivers in Hong Kong. This early intervention targeted at families with children aged 2–6 years who were suspected or diagnosed with autism spectrum disorder, developmental delay, or disabilities. The course contained nine group sessions across 2–4 months with no more than one session per week. It aimed at equipping caregivers with the skills to promote a child’s communication and learning, helping them to form a stronger relationship with the child, nurturing the caregivers’ self-care, and providing a platform for caregivers to build peer support.

### 2.2 Study design

This exploratory qualitative study was part of a larger research project that aimed to evaluate the impacts and sustainability of this localized PII. The research was conducted between August 2023 and July 2024. At the time of this research, the master trainers had trained up more facilitators in different social services units to implement this PII in Hong Kong, and the course had served many families. For this research that examined the family dynamics, we targeted the caregiver-participants who completed the PII training because they could inform us of their personal lived experiences in their families as well as their observations of the changes in their family members. Ethical approval of this research was granted by the Human Research Ethics Committee of the University of Hong Kong (reference number: EA240065).

This study was based on social constructionism and social interactionism (25). On social constructionism, we contended that our respondents’ understanding was negotiated and constructed through their experiences and their own interpretations. With new experiences disrupting the existing conceptions, they would reshape their learnings and perspectives. This philosophical approach helped us appreciate the sophistication and subjectivity of the experiences of our participants. The social interactionism approach, meanwhile, suggested that meanings were fashioned through social interactions, and this guided us to pay attention to social experiences and changing processes described by our respondents.

### 2.3 Participants

For recruitment, we partnered with the organizations that offered the localized version of WHO-CST. Target-convenience sampling method was employed. An information sheet was sent to the caregivers through our partners, and they were invited to fill in an online form if they were willing to participate in the research. Our researchers then contacted the caregivers individually to introduce the research, answer their questions, confirm their participation, and arrange them into focus groups. To recruit more participants, we also used the snowballing approach by asking the participants for referrals—friends and family members who also completed the course.

### 2.4 Data collection

Five focus groups were conducted, and each was around 1–1.5 h. Three were online discussions via ZOOM, one was physically conducted at the University of Hong Kong, and one was conducted at a social services center. Before the discussion, all participants were invited to read an online information sheet, fill in a consent form, and complete a short survey on their demographics. All participants gave us their consent to participate in this research, and all participation was voluntary. A semi-structured discussion approach was adopted, and the overarching question guiding the discussion was “Do you notice any changes in you, your child, or the people around you because of this course? What are they?” From this key discussion question, there were discussions around their experiences with the PII and the changes they observed in themselves, their children, and other family members. In total, 22 caregivers participated in these focus groups, and they were all Cantonese-speaking Chinese (Table 1). Each participant received a set of incentive package that contained a supermarket voucher costing HKD100 and a set of JC A-Connect stationeries.

**Table 1:** Characteristics of caregivers and their children.

Characteristics	No. of participants (*n* = 22)
Caregivers	
Gender identity	
Male	1
Female	21
Age	
20–29	1
30–39	15
40–49	5
50–59	1
Relationship with the child of concern	
Father	1
Mother	18
Auntie	1
Foster mother	1
Grandmother	1
Marital status	
Single	3
Married	16
Separated or divorced	3
Education	
Secondary school	9
Post-Secondary Diploma	4
Bachelor	7
Master or above	2
Employment status	
In a full-time job	5
In a part-time job	1
In multiple jobs	2
Homemaker	14
Household size	
2	1
3	9
4	5
5	5
6	1
7	1
Household monthly income	
HK$19,999 or less	3
HK$20,000 to HK$29,999	5
HK$30,000 to HK$39,999	2
HK$40,000 to HK$49,999	2
HK$50,000 or above	7
Refused to answer	3
A child who was suspected or diagnosed with developmental delays or disabilities	
Age	
2	2
3	3
4	9
5	3
6	2
7	2
8	1
Siblings	
With sibling(s) who has(have) developmental delays or disabilities	5
With sibling(s) who do not have developmental delays or disabilities	3
No sibling	14

### 2.5 Data analysis

All focus group discussion was recorded and transcribed, and the transcripts were double-checked by our research team members. Reflexive thematic analysis was chosen for its theoretical flexibility and capabilities to interact with the data to create themes from codes through an analytical and interpretative lens (26, 27). The analysis was inductive, which required us to mobilize our conceptual and design thinking when we participated in this reflexive project (28). As this study was a pioneer, we endeavored to keep ourselves open and intact to our respondents’ descriptions and perceptions. NVivo, a qualitative analysis software, was employed for coding.

Our data analysis started with our research team members first reading and familiarizing ourselves with the transcriptions. Familiarization notes were written at this stage. Next, we proceeded to open coding, then met to compare our lists of codes. Through returning to the data for further analysis, we gradually refined our codes and constructed our initial themes. These themes were reviewed in our team meetings and were reshaped and renamed to become our final themes. We further studied the relationships among the themes and constructed frameworks.

## 3 Results

**Figure 1:**
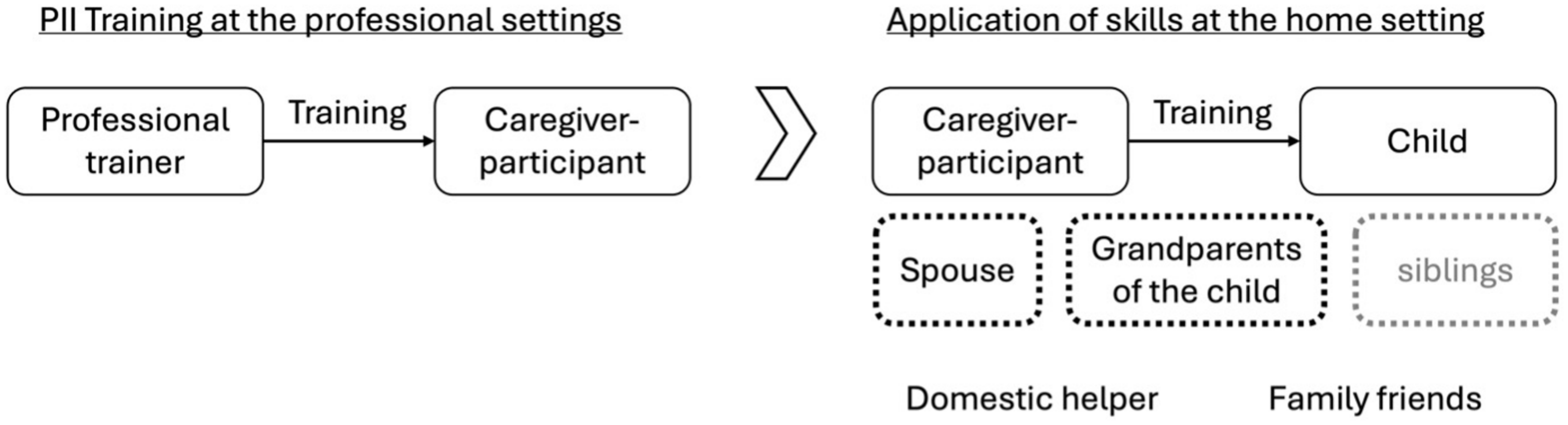
Stakeholder mapping of the PII at the professional and home settings.

Figure 1 is a stakeholder-mapping derived from our analyses. Individuals who were directly involved in the PII were presented in the solid-line boxes, while other family stakeholders—mainly spouses and grandparents of the child—who did not participate in the PII training but played a caregiving role were in dashed-line boxes. Domestic helpers and family friends were also mentioned by some respondents when they described how they implemented the PII strategies. Over one-third of our respondents had more than one child, but the interactions between the siblings were not a primary focus, as the caregiver-participants’ main concern was whether the adult family members were helpful or not.

**Figure 2:**
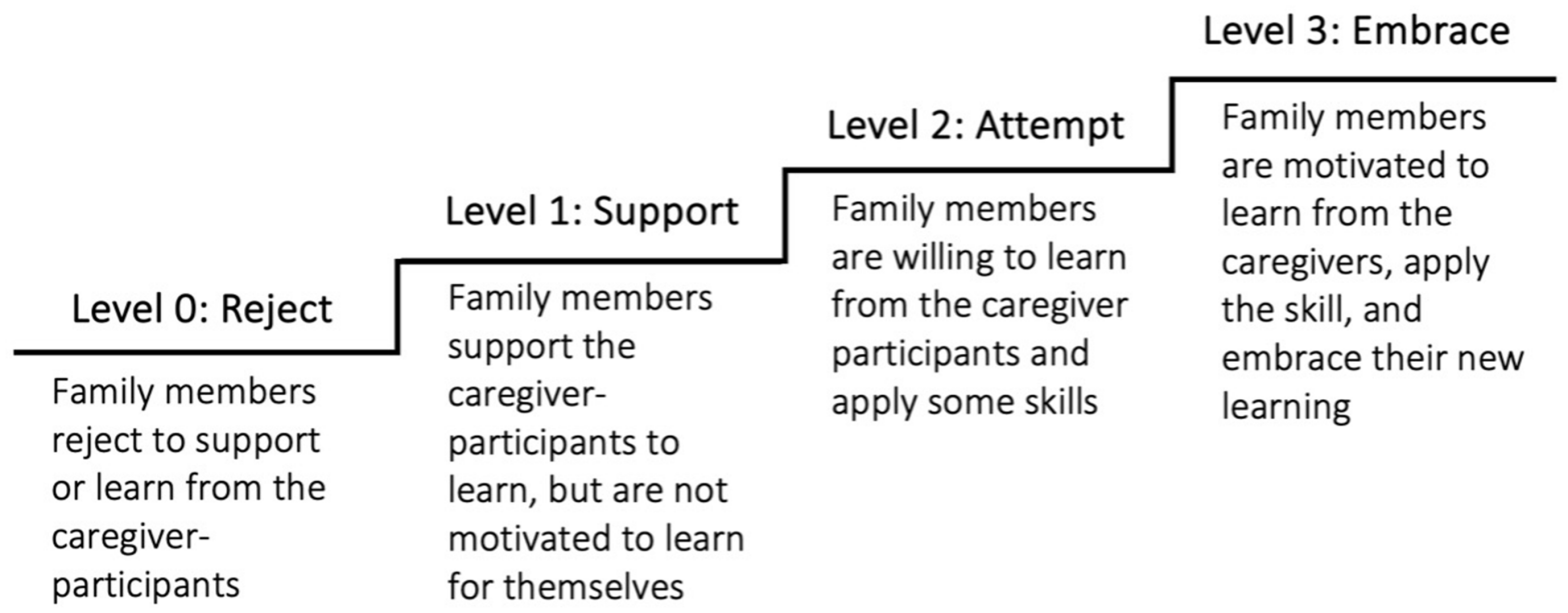
Four levels of involvement of family members who did not participate in the training of PII.

From our data, we have identified four types of responses from the adult family members, and we labelled them as Level 0: Reject, Level 1: Support, Level 2: Attempt, and Level 3: Embrace (Figure 2). These responses are presented in a stepped diagram representing a continuum of involvement. The higher the position, the higher the level of involvement of the family members in the PII.

### 3.1 Level 0: reject

For those in the *Reject* group, they were the family members who refused to support the caregivers to partake in the PII, and they also refused to learn new skills from the caregiver-participants. In most cases, this stemmed from the belief that underperformance in some children might happen, and it would be resolved over time naturally as they grew up. This normalization of the presenting symptoms led family members to overlook the root causes. When no problem was recognized, no solution was needed.

*He (Husband) still does not accept that his son has autism spectrum disorder (ASD). He wonders: Why did the doctor say so? My son is so normal. After he listened to the report, he said, “Who cares about ASD?” It is completely difficult for him to learn from me. (C16).*

Some family members holding this perspective were not only downplaying the problems, but they even discouraged the caregiver-participants to acquire knowledge about special educational needs, suggesting that their worries were unnecessary. This disapproval added to the caregivers’ burden.

*My husband and my mom are relatively stubborn, so it is difficult (to share with them). Also, we do not have much knowledge about children with special needs… They do not see the needs to learn because they assume that the child will get better by himself, this is a mainstream perspective. “You do not need to do much, you are giving yourself too much pressure…” When family members are not willing to learn, my pressure is tremendous. (C05).*

Family members were not always negative. Some rejected to support because they wanted to insist on their own parenting style.

*I live with the four grandparents of the child, they still insist in their own ways, so it is difficult. (C12).*

Some family members did not seem to care because the family culture upheld independence. A mother shared that although she lived with the paternal and maternal grandparents of the child, each family unit was expected to maintain distinct boundaries and not to intervene in others’ interactions with the child; hence, it was natural for them not to offer any support.

*We (The young family with a child and the grandparents of the child) are like housemates sharing the same apartment, we each take care of our businesses. They (grandparents) do not involve in my parenting, and I do not get involved in their communication with my child.*

In our focus group, this respondent further explained that her choice of keeping the learning to herself was her way of paying respect to the elders. Some caregiver-participants also held this belief, leading them to avoid sharing with family members deemed unlikely to be receptive.

*Each of us has our own communication style and perspectives… Making the older generation to learn is difficult, so why not allowing them to explore and live peacefully? (C13).*

The family members falling in the *Reject* group were the least helpful to the caregiver-participants. Fortunately, they were the minorities.

### 3.2 Level 1: support

More family members belonged to the *Support* group, who were willing to support the caregivers to acquire new skills, yet they lacked motivation to learn for themselves. A mother shared that her husband initiated to provide support, such as taking leaves to care for the child so she could participate in the course; nevertheless, he was not motivated to learn.

*My husband does not proactively learn these things. He does not see that it is your full responsibility because you have acquired the skills, he is just not into learning. He is willing to take care of the child. When I share the new learning with him, he listens, and this is it… It is only me who learns. My husband would ask, “Do you need me to take a leave? When you attend classes, I can take a leave and stay at home with our child so that you can concentrate on learning.” I find this helpful. (C01).*

Busyness at work was another reason hindering the supportive family members to learn new skills.

*My husband, to some extent, is very helpful. However, he did not participate in the course and is very occupied, he cannot use the skills. (C04).*

While the caregiver-participants appreciated the support provided by their partners, they were hoping that they would be open to learn to align their parenting styles. They found that when their approaches were unaligned, this could be counterproductive.

*If a mom learns the skills, but the dad has no knowledge about them; after you train your child then your husband does the opposite, then all the efforts are in vain. (C01).*

The caregiver-participants with family members at *Level 1: Support* were, in general, grateful for the help they received, and they appeared to have better psychological wellbeing than those with families in the *Reject* group.

### 3.3 Level 2: attempt

Most of the caregiver-participants reported having family members who are open to learn and trying out some skills. Some family members who were aware of the needs of the child were curious about the skills and initiated to ask and learn.

*Grandpa (Father of the caregiver-participant) always helps take care of my daughter, as now he has retired. When he sees his grandchild, he always asks, but he is unable to apply the skills well… To some extent, they (grandparents) understand there are things to observe that would help the child, they are interested and want to know more. Though they are not yet competent to use the skills, especially using several skills simultaneously, they have the sense that there are ways to support the child (C20).*

In most cases, it was the caregiver-participants who were proactive in sharing and demonstrating the skills for their family members.

*My husband has no time because I have now become a full-time caregiver. However, when we are together, I try my best to share with him, and I hope he would adopt my parenting style. He imitates how I communicate with the child and tries to remember. (C15).*

One of the successful strategies facilitating skill acquisition was to assign family members specific tasks with clear instructions and well-defined steps.

*Sometimes when grandma takes care of the child, I would assign her some tasks. It is great that grandma is willing to follow the clear steps that I listed. We work together to help the child. I also translate some of my learning to share with my domestic helper at home. She has some friends who also care for children with ASD and they share with one another about how to care for these children. (C08).*

Some family members, who once refused to acknowledge the challenges that the child faced, were gaining new insights into how a parenting approach influenced a child’s emotional, behavioral, and social development when the caregiver-participants explained to them. They were then willing to apply some suggested alternative practices.

*Grandpa is the primary caregiver, and grandma needs to work. Grandpa does not really accept that his grandson could have some difficulties. He tends to spoil him. The child did not need to speak, grandpa observed and gave the child what he wanted. We explained to grandpa what the problems were. Now, he sometimes waits for the child to request to provide for him. (C22).*

More than half of the caregiver-participants reported that their family members exhibited positive changes when they attempted to apply the techniques. These changes included being more supportive in caregiving and experiencing more positive emotions.

*When I found the new skills useful, I would share with my husband. Now he is participating more (in parenting), really. In the past, he did not seem to care about his son as if it was none of his business. Now it is much better. (C10).*

*I do meditation (a learning from the PII) with my husband. I lead him to do it. I can see that his emotions are a lot better when he relates with the child. He observes me and learns some skills on communicating with a child. He has changed a lot. (C11).*

The caregiver-participants with family members at *Level 2: Attempt* were more positive about the outlook for the child’s development. They felt supported by their family members and were motivated to share their learning as their families were receptive. They also took pride in their own efforts in creating changes to the family dynamics.

### 3.4 Level 3: embrace

Three caregiver-participants excitedly shared about the transformation of their family members who now embraced the skills. These were usually the husbands who did not know how to handle their children but have now developed a much closer and satisfying relationship when they applied their new learning. Unlike those at *Level 2: Attempt* who were willing to learn and apply a few techniques, these family members were owning their learning and applying their new skills without the prompting from the caregiver-participants.

For family members who reached *Level 3: Embrace*, they usually went through a transformation process which started from being inspired by the usefulness of the strategies. The point of intervention made a difference in promoting the family members’ motivation to learn. The opportune moments came when the family members were facing difficult situations, and the caregiver-participants were able to demonstrate how the skills could effectively solve the parenting problems. The family members would be surprised and hooked by the new solutions.

*My husband was bringing the child to the playroom. Usually, the child would insist to continue to play, and my husband would have no choice but to forcefully carry him back. The child would then become emotionally unstable and resisted to do his homework, taking a shower or eat. That day my husband called me and passed me the phone. I said, “You like playing at the playroom, do not you?” … (After the call), the child passed the phone to my husband saying, “grandpa, let us go.” When they were back home, we were so surprised about him being cooperative. (C17).*

Being able to experience positive outcomes from applying the skills served as an additional motivator for the family members. The positive experiences fueled their desire to learn more, and this formed a positive reinforcement cycle.

*What did I teach him? For example, I told my husband that he did not need to hold the kid all the time. He can hold him, count to ten, and put him down… Now my husband carries him and counts to ten. What I have learnt, I also want my husband to apply… he thinks the skills are useful, so he is willing to learn. (C14).*

A grandmother *(C17)* further shared that when her husband could now go along with his grandson, he could share more caregiving role, and she felt more relieved as she now had more time for herself.

*My husband used to give him extra $2, I told him many times (not to). After I attended this course, I explained to him that if you and I aligned, this would help the child and me, it would make my caring role a lot easier. He listened, understood and followed, very cooperative. Now the child likes him more than me. In the past, the child would insist that three of us must go together. Now he leaves me alone and spends time with his grandpa. My husband enjoys being with him a lot. He finds that he can also have a satisfying relationship with his grandson. (C17).*

Praises and affirmation also helped bolster the family members’ confidence in applying the skills. A mother *(C08)* shared that aside from assuring her husband of his capabilities from time to time, she also invited her friends to her home and assigned them tasks to praise her husband to supercharge his sense of competence.

*He used to tell the child to find me every time as he thought he could not manage his grandson. I said, “you can (take care of the child), we all can. You need to be willing to try and spend more time with him.” Now when the child wants to play, he prefers him to me. (C17).*

*I am lucky to have friends so support me. I invite them to help. Some would praise my husband for doing a better job than me that he had more potentials to be a trainer. He then started to believe that he could make it. (C08).*

As the family members embraced their learning, they were willing to take extra steps. A mother shared that her husband had turned from being hesitant to accept his fathering role to now desiring to spend time to play with his son whenever he could.

*My husband did not like to learn so I summarized the key lessons to share with him. I would assign him the role of training our child and giving him the responsibility because he could not relate with the child. He was willing to learn but I think he did not know how to engage a child. After I took the course, my husband’s relationship with the child has improved a lot. When he comes home after work and the child still has not slept, he would spend half an hour to play with him. Lately, he could finally bring our kid to the park by himself. (C08).*

**Figure 3:**
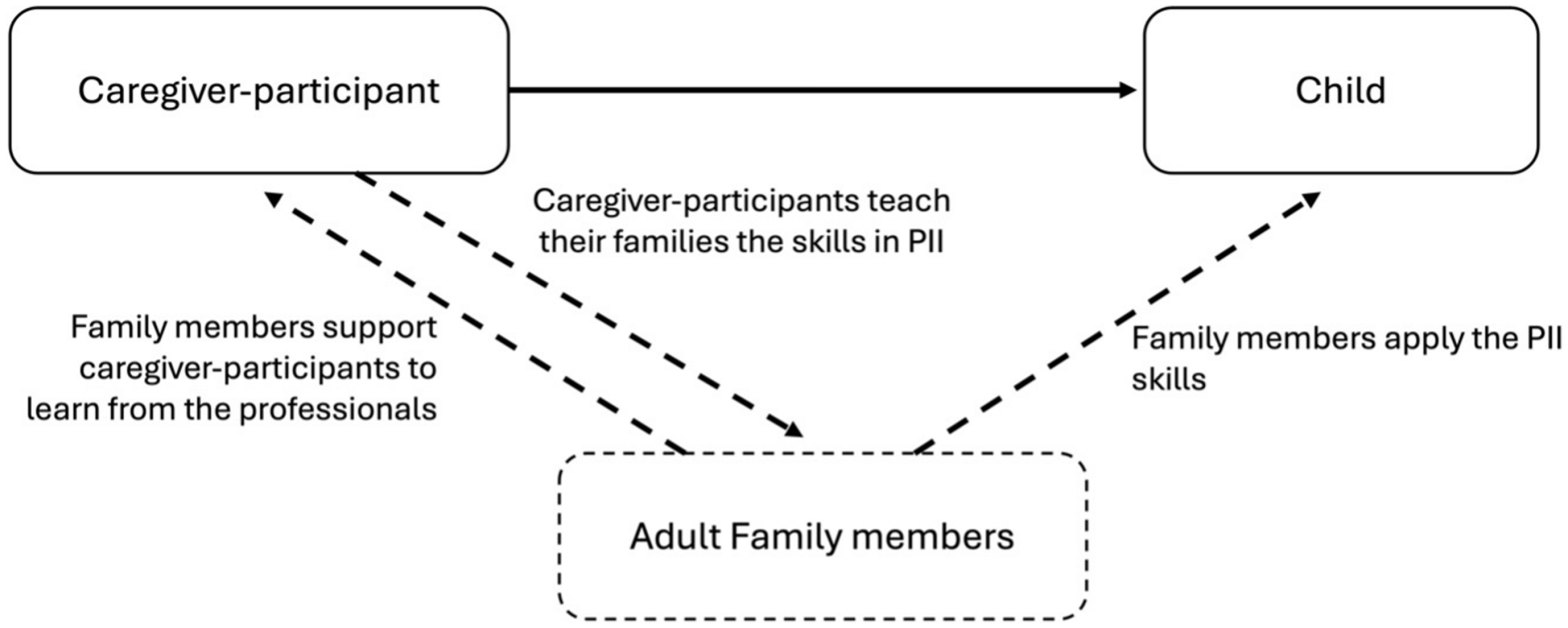
Support in families.

When we followed up on these triumphant stories, we found that the family members who were now at *Level 3: Embrace* were once at *Level 1: Support* or *Level 2: Attempt*. In our dataset, we did not have caregiver-participants sharing about their very motivated family members initiating to learn and embracing their learning instantly (This could be the case in other research). The changes were progressive with the caregiver-participants’ continuous support and reassurance. Figure 3 is a simplified presentation summarizing the support in families.

## 4 Discussion

This research aimed at exploring how family members who did not participate in the PII training were influenced by and influencing the intervention at home. When the earlier review and conceptual paper urged the practitioners to examine how PII functions as a family-centered approach, this study responded to the call to look into the family dynamics (13, 29, 30). With the caregiver-participants’ description of the reactions of their family members—mainly the spouses and grandparents of the child who assumed the caregiving roles—we identified four types of responses. These responses had a particular focus on whether the adult family members were willing to get involved in the learning and practice of PII. This focus was steered by and commonly shared among the caregiver-participants. This was what they cared about most when family members were of concern. In this discussion, the caregiver-participants also revealed how these responses influenced their wellbeing and their use of skills.

**Figure 4:**
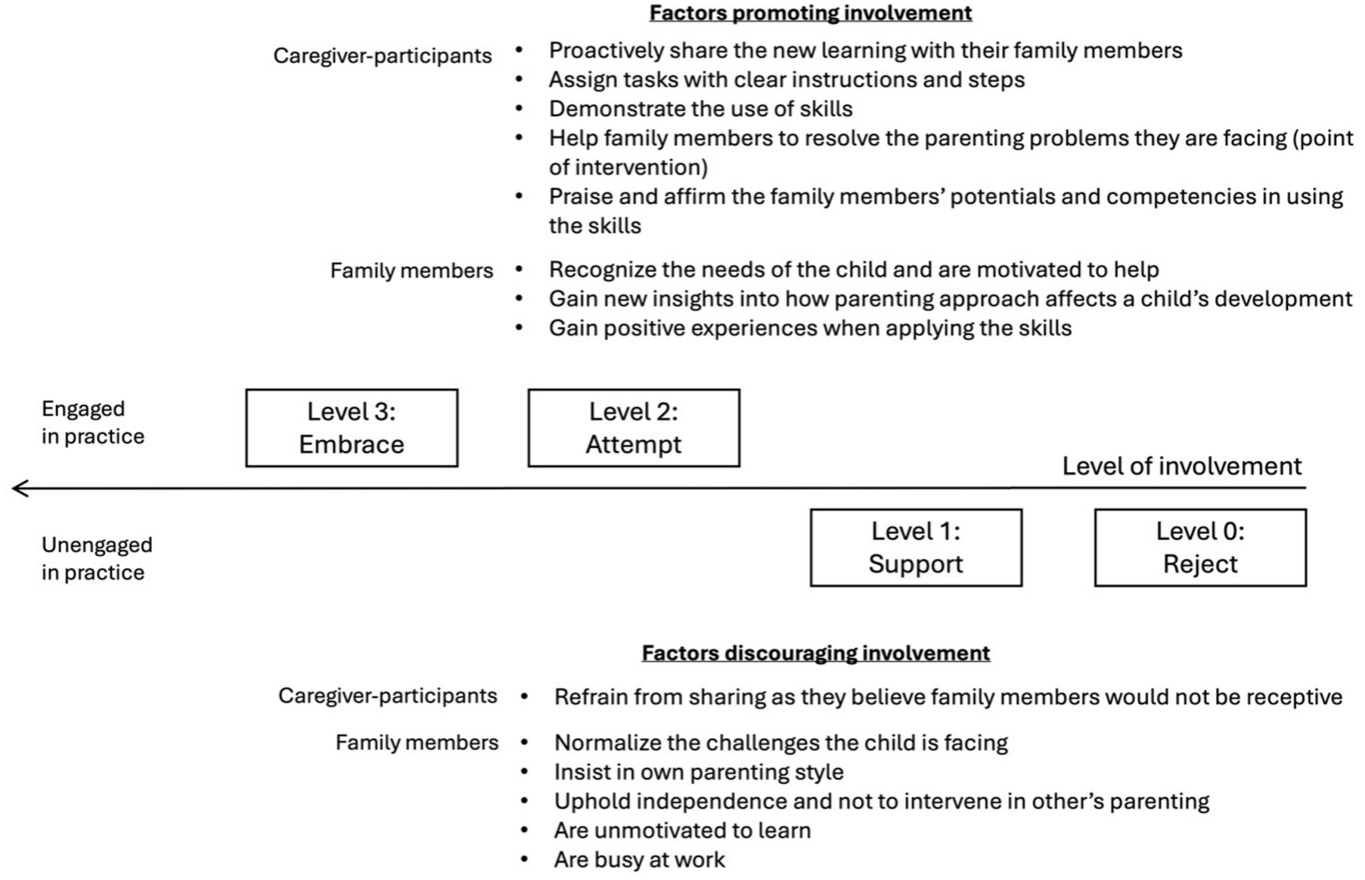
Factors affecting family members’ involvement in the practice of PII.

The four types of responses are on a continuum of family members’ level of involvement in the practice of PII. The higher the level, the higher the involvement. Figure 4 summarizes the factors affecting family members’ involvement.

At *Level 0: Reject*, family members were not supportive of caregivers’ learning and application of PII. This non-engagement stemmed largely from their normalization of the child’s developmental delay, for they believed that the child would naturally pick up the essential skills, such as speaking and playing with others. Other reasons contributing to their non-participation included their insistence on own parenting style and the family culture that valued respecting other people’s parenting styles. In such cases, the caregiver-participants chose not to share their learning with their family members. Another quantitative research study also discovered negative feedback from the family members, as they complained that the caregiver-participants focused mainly on the child of concern and neglected their spouses and other children. However, we did not collect such feedback in this research (30). *Level 0: Reject*, caregiver-participants were feeling lonely and stressed as they were not supported and understood.

At *Level 1: Support*, family members were much more welcoming towards the PII. They were willing to share the caregiving roles or even take up more to allow the caregiver-participants room to attend the classes. While they were being supportive, they were unmotivated to learn for themselves, as they were busy or simply not interested in learning. Some caregiver-participants tried to share their learning, but their family stakeholders were not receptive. Caregiver-participants with this type of family member were generally grateful for the loving support. Nevertheless, they were hoping that other caregivers in the families would be more open to learn the new skills so that their parenting styles could align and bring about the best outcomes for the child. *Level 1: Support* is a step-up when compared with *Level 0: Reject*, but both types of family members were unengaged in the practice of PII.

At *Level 2: Attempt*, family members were more willing to learn from the caregiver-participants and apply some skills. Some initiated to learn because they recognized the needs of the child and wanted to help promote the child’s development. Some were originally reluctant to accept the child’s problems, but were gaining insights after the caregiver-participants explained to them. For family members to try out the skills, the caregiver-participants, in many cases, played a crucial role in proactively involving them in the practice of PII. Aside from sharing their learning, caregiver-participants also assigned other caregivers tasks with clear instructions and procedures so that they could be implemented. Caregiver-participants with family members at *Level 2: Attempt* felt more supported and hopeful. They were witnessing the changes in their children and their family members, and took pride in the positivity they introduced to the family system.

At *Level 3: Embrace*, family members were owning their new learnings, putting them into practice without the nudge from the caregiver-participants, and were building a much closer and positive relationship with the child. In our data, it was mainly the husbands who did not know how to relate to their child and now became confident to take up the caregiving role independently. We found three motivators for this transformation. First, the caregiver-participants were helping their family members to solve the parenting challenges they were facing, and successfully demonstrated the new skills. When the caregiver-participants strategically chose this point of intervention and the family members found the skills useful, they were enthused to learn. Second, when the family members put the skills into practice and achieved positive outcomes, the positive experiences then fueled their desire to learn more, thus creating a positive reinforcement loop. Third, the caregiver-participants intentionally and constantly praised their spouses or even involved people around them to affirm their husbands’ competencies. This boosted their confidence to continue to practice and transform. Caregiver-participants with family members at *Level 3: Embrace* were the most satisfying ones. They applauded the achievements of their partners, received more support from them, enjoyed better relationships in families, and were more relieved as their partners could share more caregiving tasks.

For our respondents, most of their family members belonged to the *Level 2: Attempt* group, some were in the *Level 1: Support group*, and a few were in the *Level 0: R*eject and *Level 3: Embrace* groups. It was common to have a mix of different types in a family.

Although this research is limited by its small sample size and the lack of direct involvement of family members in our data collection, it offers us valuable insights into the needs of the caregivers, the challenges they face at home, and the joy they found when other caregivers were willing to support and participate in PII.

## 5 Implications and future directions

The current study has confirmed the necessity for stretching the scope of examination from focusing entirely on the changes of the caregiver-child dyads to the changes of the indirect stakeholders—the family members who did not participate in the PII training. As we depicted the reactions of these family members, our findings revealed that PII had indeed introduced changes to the family systems. From what we observed, a majority of the family members were willing to acquire some skills to support the caregiver-participants and the child, and this reshaped the parenting approaches in the families. Evidence supported that the impacts of PII were beyond the intended outcomes of the programs. On this note, we recommend more research to explore the rolled-on effects of PIIs. These effects could be on families, organizations that offer the PII trainings, and the communities. To support such work, Figure 1 offers a graphical presentation of the stakeholder mapping, and this flowchart can be used for analyzing the systems around PII.

Furthermore, when we examined how family members’ responses affected the caregiver-participants, we found that their reactions inevitably influenced the wellbeing of the caregiver-participants and their practice of skills at home. With unsupportive family members, the caregiver-participants faced more hindrances in putting the skills into practice and felt lonelier. With supportive members, the caregiver-participants thrived. When other caregivers were unwilling to learn, the misalignment of parenting styles could reduce the effects of PII. Conversely, if they welcome the new practices and bring them into use, the effects of PII multiplies. Hence, this research showed that by shedding light on the changing processes and outcomes in families, it helped not only to delineate a more comprehensive picture of the impacts of PIIs, but it also helped map out the familial factors affecting the effectiveness of the programs. The existing literature suggested that the design of PIIs should consider the family’s characteristics (29) and that the intervention could stretch to promote the family’s functioning (31). This work helped pave the way. For designing a PII, practitioners are encouraged to use the framework in Figures 2, 3 as roadmaps to consider the social exchanges in families that could affect the program’s efficacy. For academics or professionals who seek to examine the risk and protective factors in families, Figure 4 provides an initial framework for such investigation. Future research may examine how background characteristics affect family outcomes. This area has been initially explored in this study; however, no conclusive cause-and-effect relationships can be established given our small sample size.

Moreover, as we delved into our respondents’ stories to see how they perceived the changes of other caregivers, we recognized that PII was not merely a project of learning some skills to support the child. For some caregivers, it was also their endeavor to rewrite their family dynamics and trajectories. As shown in our results and discussion, the intentionality of the caregivers to involve their family members was a key driver of changes. For caregiver-participants, Figure 4 offers some insights into the good practices that they can adopt to promote familial changes. Furthermore, as this research discovered that family stakeholders could change over time—some family members gained new insights after the caregiver-participants explained to them, and caregivers could proceed from *Level 1: Support* to *Level 2: Attempt* to *Level 3: Embrace*—it offers encouragement to the caregiver-participants to be persistent in their efforts as people take time to change.

To accumulate more empirical evidence to strengthen the design and use of PIIs, we suggest conducting more qualitative studies to delineate the changing processes in families and identifying factors affecting the changes. With our small sample size, we make no claims of arriving at a comprehensive list, but our findings could serve as a starting point for future exploration. Conducting such research in different contexts (e.g., different cultures, different PII interventions) can also provide us with patches of new knowledge. When we have more sets of data for comparison, we can then weave them together to identify patterns and conduct quantitative studies to build new models. Our research seeks to provide a foundation for these future meaningful endeavors.

## 6 Conclusion

With developmental delay and disabilities becoming more of a public health concern, PII has been a valuable early intervention that supports the development of children and their families. When PII is designed to be implemented at home, it is important to explore how it affects the family dynamics and how the family systems affect the effectiveness of the intervention. As we strive to bridge the gap of lacking understanding of PII in a family context, we seek to construct frameworks that support the future design of PIIs and the PII-related research.

## Data Availability

The Datasets presented in this article are not readily available because of the confidentiality agreement between the researchers and the participants but are available from the corresponding author on reasonable request. Requests to access the datasets should be directed to PW, paulw@hku.hk.
